# Impact of fully automated assessment on interstudy reproducibility of biventricular volumes and function in cardiac magnetic resonance imaging

**DOI:** 10.1038/s41598-021-90702-9

**Published:** 2021-06-02

**Authors:** Sören J. Backhaus, Andreas Schuster, Torben Lange, Christian Stehning, Marcus Billing, Joachim Lotz, Burkert Pieske, Gerd Hasenfuß, Sebastian Kelle, Johannes T. Kowallick

**Affiliations:** 1 University Medical Center Göttingen, Department of Cardiology and Pneumology, Georg-August University, Göttingen, Germany; 2 German Center for Cardiovascular Research (DZHK), Partner Site Göttingen, Göttingen, Germany; 3 Philips Healthcare, Hamburg, Germany; 4 University Medical Center Göttingen, Institute for Diagnostic and Interventional Radiology, Georg-August University, Robert-Koch-Str. 40, 37075 Göttingen, Germany; 5 Department of Internal Medicine and Cardiology, Charité University Medicine, Campus Virchow Klinikum, Berlin, Germany; 6 German Heart Center Berlin, Berlin, Germany

**Keywords:** Cardiology, Characterization and analytical techniques

## Abstract

Cardiovascular magnetic resonance (CMR) imaging provides reliable assessments of biventricular morphology and function. Since manual post-processing is time-consuming and prone to observer variability, efforts have been directed towards novel artificial intelligence-based fully automated analyses. Hence, we sought to investigate the impact of artificial intelligence-based fully automated assessments on the inter-study variability of biventricular volumes and function. Eighteen participants (11 with normal, 3 with heart failure and preserved and 4 with reduced ejection fraction (EF)) underwent serial CMR imaging at in median 63 days (range 49–87) interval. Short axis cine stacks were acquired for the evaluation of left ventricular (LV) mass, LV and right ventricular (RV) end-diastolic, end-systolic and stroke volumes as well as EF. Assessments were performed manually (QMass, Medis Medical Imaging Systems, Leiden, Netherlands) by an experienced (3 years) and inexperienced reader (no active reporting, 45 min of training with five cases from the SCMR consensus data) as well as fully automated (suiteHEART, Neosoft, Pewaukee, WI, USA) without any manual corrections. Inter-study reproducibility was overall excellent with respect to LV volumetric indices, best for the experienced observer (intraclass correlation coefficient (ICC) > 0.98, coefficient of variation (CoV, < 9.6%) closely followed by automated analyses (ICC > 0.93, CoV < 12.4%) and lowest for the inexperienced observer (ICC > 0.86, CoV < 18.8%). Inter-study reproducibility of RV volumes was excellent for the experienced observer (ICC > 0.88, CoV < 10.7%) but considerably lower for automated and inexperienced manual analyses (ICC > 0.69 and > 0.46, CoV < 22.8% and < 28.7% respectively). In this cohort, fully automated analyses allowed reliable serial investigations of LV volumes with comparable inter-study reproducibility to manual analyses performed by an experienced CMR observer. In contrast, RV automated quantification with current algorithms still relied on manual post-processing for reliability.

## Introduction

Cardiovascular magnetic resonance (CMR) imaging is the reference standard for the assessment of cardiac morphology and function^1,2^, amongst which left ventricular ejection fraction (LVEF) is most commonly used for cardiovascular risk assessment and clinical decision making^3,4^. Compared to echocardiography, CMR demonstrates superior inter-study reproducibility resulting in considerably lower sample sizes required to show clinically relevant changes in left ventricular (LV) and right ventricular (RV) dimensions and function^5,6^. However, in clinical routine CMR requires extensive post-processing, which is time-consuming, tedious and prone to observer variability^7–10^. Despite efforts directed towards automation of volume and mass assessments, most approaches require manual preparation and preselection of CMR images^11,12^. More recently, novel artificial intelligence (AI)-based deep learning algorithms were introduced which allow for fully automated post-processing of LV mass and biventricular volumes showing promising initial results including risk stratification following acute myocardial infarction^13,14^. Data on interstudy reproducibility is of high clinical importance when it comes to follow-up surveys. Observer experience and variability may significantly impact the identification of subtle clinical changes between exams^10^. Hence, the current study aimed to assess the impact of fully automated assessments on inter-study variability and reliability in comparison to an experienced and inexperienced observer to define the current potential and limitations of fully automated post-processing.

## Methods

### Study population

The study population consisted of 18 participants which were scanned twice at a median interval of 63 days (range 49–87) using a standardized imaging protocol for anatomy and function^15,16^. All participants were in stable sinus rhythm during image acquisition. A minimum of 6 weeks between the first and second scan was required to avoid recollection bias of the involved CMR staff. Care was taken that acquisitions were performed at the same levels of the heart. Care was taken that no change in symptoms and medication occurred in patients with heart failure. Furthermore, new onset of cardiac disease was excluded in healthy subjects. The study was approved by the Ethics Committee of the Charité-University Medicine Berlin and was conducted according to the principles of the Helsinki Declaration. All participants gave written informed consent before randomization. The study was supported by the German Centre for Cardiovascular Research (DZHK).

### Cardiovascular magnetic resonance imaging

Electrocardiogram (ECG)-gated balanced steady-state free precession (bSSFP) cine images were acquired in 10–16 equidistant short axis (SA) planes covering both entire ventricles on a clinical MR scanner (1.5 T, Achieva, Philips Healthcare, Best, The Netherlands). Imaging parameters were as follows: 25 frames/cardiac cycle, pixel spacing 0.8 mm × 0.8 mm, 8 mm slice thickness as well as inter-slice gap, TE 1.5 ms, TR 3 ms.

**Figure 1:**
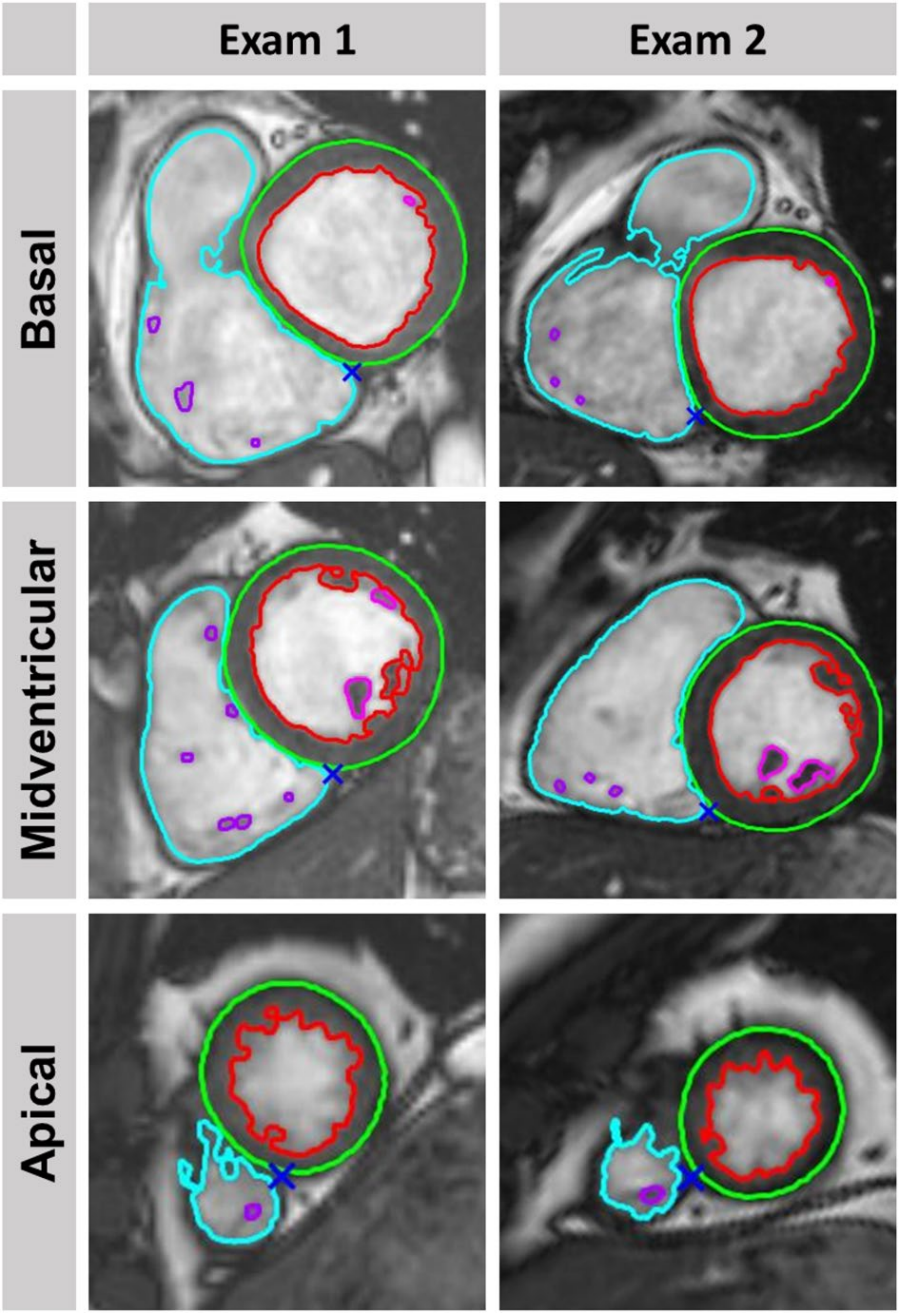
Fully automated biventricular segmentation. The figure depicts automated biventricular volume assessments for a representative volunteer at the basal, midventricular and apical level at baseline MRI (Exam A) and follow-up MRI (Exam B). Higher inter-study variability may potentially be induced by the basal segmentation in the example.

Manual volumetric assessments were performed in SA orientations according to standardized recommendations^17^ by an experienced CMR operator (observer A, cardiologist, 3 years of CMR experience) and an inexperienced operator (observer B, trainee in cardiology, no experience in reporting or CMR segmentation), who was trained 45 min by the experienced observer with five cases from the SCMR consensus data^18^. Long-axis views (4-chamber and 2-chamber) were crosslinked to define RV and LV basal segments. Dedicated commercially available post-processing software was employed for manual assessments (QMass, Version 3.1.16.0, Medis Medical Imaging Systems, Leiden, The Netherlands). Fully automated analyses were performed in SA stacks with suiteHEART (Version 4.0.6, Neosoft, Pewaukee, WI, USA), Fig. 1. Papillary muscles were included within the myocardium. Fully automated analyses were not manually post-processed or validated, manual segmentations were not supported by any semi-automated processing e.g. threshold or edge detection. All operators were blinded to their previous as well as each other’s results. Volumetric analyses comprised LV mass, LV and RV end-diastolic/systolic (EDV/ESV) volumes as well as stroke volumes (SV) and EF. Interstudy agreements were evaluated for manual assessment of observer A, manual assessment of observer B as well as fully automated analyses.

### Statistical analyses

Statistics were calculated using IBM SPSS Version 24 for Windows (IBM, Armonk, NY, USA) and Microsoft Excel. Continuous parameters are reported as mean and corresponding standard deviation (SD), changes from Exam 1 to 2 were evaluated using the Wilcoxon signed-rank test for dependent continuous parameters. An alpha level of 0.05 and below was considered statistically significant. Inter-study and inter-observer variability was assessed using intra-class correlation coefficients (ICC) based on absolute agreement (excellent ICC > 0.74, good between 0.60 and 0.74, fair between 0.4 and 0.59 and poor below 0.4)^19^, the coefficient of variation (CoV, SD of mean difference divided by the mean (SD (MD))/mean) as well as Bland–Altman plots [mean difference between measurements with 95% confidence interval (CI)]^20^. Intra-observer reproducibility of the automated algorithm has been addressed previously yielding ICC = 1 and CoV 0%^13^. Sample sizes were calculated for the detection of absolute changes of 10 g LV mass, 10 ml LV and RV EDV/ESV/SV as well as 5% change in LV/RV-EF for a power of 80% and an α-error of 0.05 using the formula $n=f \left(\alpha , P\right)*{\sigma }^{2}*\frac{2 }{{\delta }^{2}}$ where n = sample size, f = factor taking α (level of significance) and P (study power) into account (f = 7.85 for α = 0.05 and P = 0.8), σ = interstudy standard deviation of the mean difference between Exam 1 and 2 and δ the magnitude of differences to be detected^5,6^.

## Results

### Study population

The study population consisted of 18 participants, 11 with normal biventricular function and 7 with heart failure, the latter including 3 patients with heart failure and preserved (HFpEF) and 4 patients with reduced (HFrEF) ejection fraction. The mean age was 46 years with a SD of 23. Ten participants were male and 8 female. All SA stacks were assessed by observers A and B as well as by the fully automated software algorithm. Results for LV and RV volumes are reported in Table 1. LV volumes and function were not significantly different between exams 1 and 2 for observer one and two as well as automated analyses. Statistically significant differences in RV volumetry were observed for observer A and the automated software algorithm reported in Table 1. Manual post-processing took on average 8.5 ± 1.7 min and 13.2 ± 2.8 min for the experienced and inexperienced observer, as opposed to automated analyses with < 1 min/SA stack.

**Table 1:** Cardiac volumes.

	Exp Exam 1	Exp Exam 2	*p*	Inexp Exam 1	Inexp Exam 2	p	Automated Exam 1	Automated Exam 2	*p*
LV Mass (g/m^2^)	104 ± 35	102 ± 34	0.184	101 ± 27	104 ± 36	0.879	103 ± 39	100 ± 36	0.088
LV EDV (ml/m^2^)	176 ± 55	173 ± 51	0.267	162 ± 44	171 ± 57	0.199	170 ± 50	173 ± 56	0.500
LV ESV (ml/m^2^)	84 ± 46	82 ± 40	0.396	75 ± 37	81 ± 47	0.215	80 ± 42	81 ± 45	0.557
LV SV (ml/m^2^)	91 ± 21	91 ± 20	0.679	87 ± 24	90 ± 20	0.327	90 ± 19	92 ± 18	0.316
LV EF (%)	54 ± 11	54 ± 9	0.744	55 ± 11	55 ± 11	0.601	55 ± 10	56 ± 9	0.499
RV EDV (ml/m^2^)	140 ± 29	143 ± 30	0.064	155 ± 39	161 ± 45	0.122	153 ± 37	161 ± 44	**0.045**
RV ESV (ml/m^2^)	56 ± 18	55 ± 16	0.500	82 ± 25	84 ± 27	0.711	67 ± 23	75 ± 27	**0.011**
RV SV (ml/m^2^)	84 ± 15	88 ± 17	**0.039**	73 ± 23	77 ± 23	0.472	86 ± 19	85 ± 25	0.523
RV EF (ml/m^2^)	60 ± 7	62 ± 5	**0.043**	47 ± 9	48 ± 8	0.811	57 ± 7	53 ± 8	**0.041**

Cardiac volumes as assessed manually by the experienced (exp) and inexperienced (inexp.) observer as well as fully automated. Volumes are reported as mean ± standard deviation. LV mass is reported in gram, volumes in ml and EF in %.

*P*-values in bold type indicate statistical significance < 0.05.

*LV* left ventricular, *RV* right ventricular, *EDV* end-diastolic volume, *ESV* end-systolic volume, *SV* stroke volume, *EF* ejection fraction.

### Reproducibility

**Figure 2:**
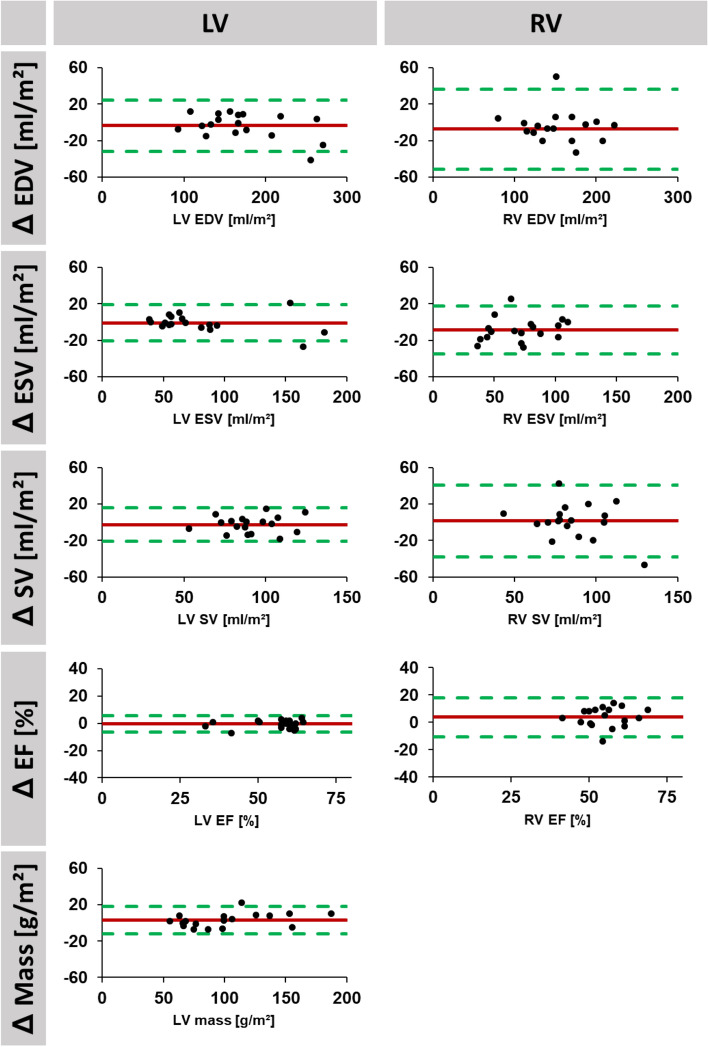
Agreement of short axis volume assessments based on fully automated analyses. Bland Altman plots are shown for interstudy reproducibility of left (LV) and right (RV) ventricular end-diastolic (EDV) and -systolic (ESV) as well as corresponding stroke volume (SV) and ejection fraction (EF). LV assessments also included LV mass. (Δ = difference for interstudy measurements. Red: bias; green: limits of agreement.

**Figure 3:**
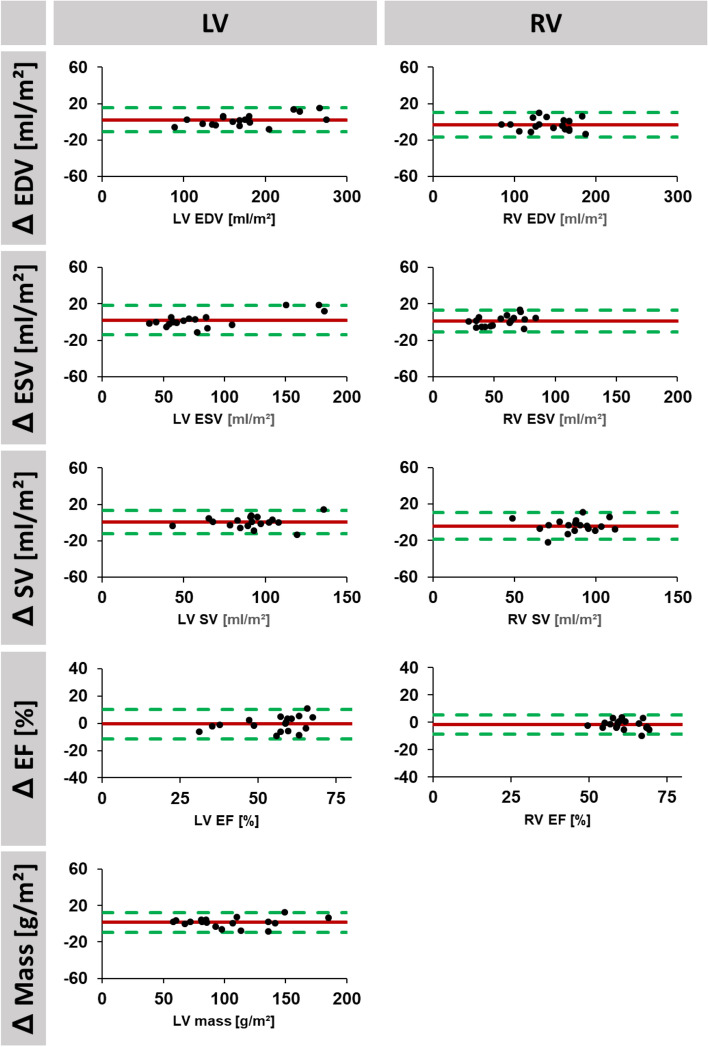
Agreement of short axis volume assessments based on the experienced observer. Bland Altman plots are shown for interstudy reproducibility of left (LV) and right (RV) ventricular end-diastolic (EDV) and -systolic (ESV) as well as corresponding stroke volume (SV) and ejection fraction (EF). LV assessments also included LV mass. (Δ = difference for interstudy measurements. Red: bias; green: limits of agreement.

**Figure 4:**
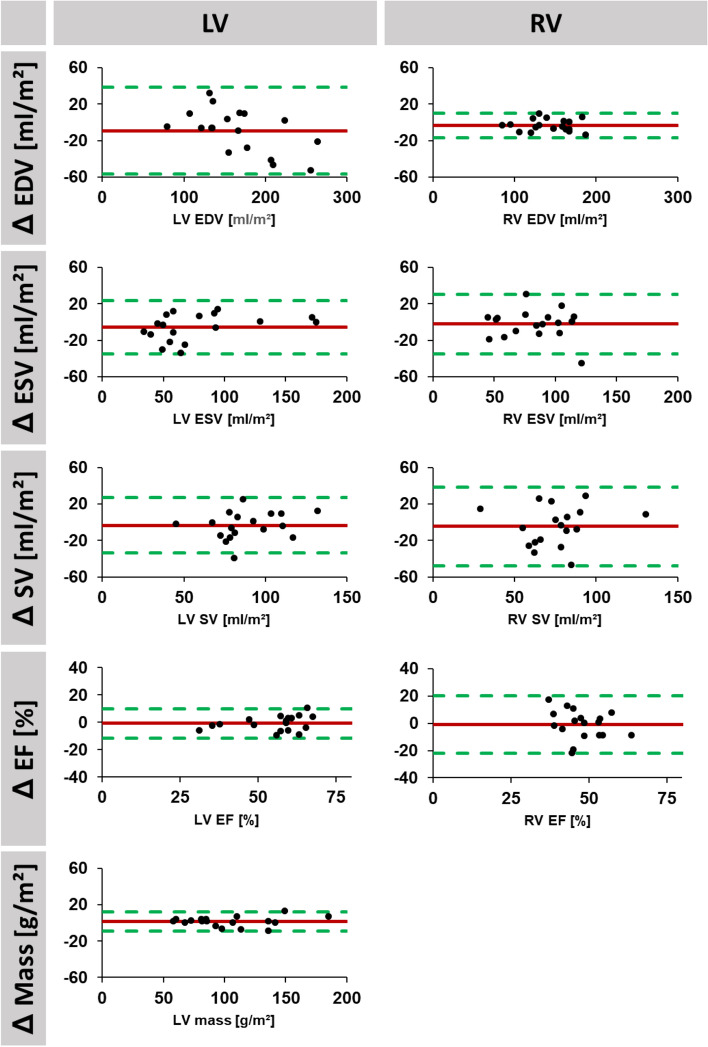
Agreement of short axis volume assessments based on the inexperienced observer. Bland Altman plots are shown for interstudy reproducibility of left (LV) and right (RV) ventricular end-diastolic (EDV) and -systolic (ESV) as well as corresponding stroke volume (SV) and ejection fraction (EF). LV assessments also included LV mass. (Δ = difference for interstudy measurements. Red: bias; green: limits of agreement.

For interstudy reproducibility, mean differences as well as corresponding SD, ICC and CoV of LV and RV volumes are reported in Table 2, corresponding Bland–Altman plots are displayed in Figs. 2, 3 and 4. LV reproducibility was overall excellent (ICC 0.86–1.00), best for observer A (ICC > 0.98), followed by fully automated analyses (ICC > 0.93) and observer B (ICC > 0.86). Interstudy reproducibility of RV volume was excellent for observer A (ICC > 0.88), good to excellent for automated analyses (ICC 0.69–0.92) and fair to excellent for observer B (ICC 0.46–0.95). Similarly, lowest interstudy variability was found in LV volumes for observer A (CoV < 9.6%) followed by fully automated analyses (CoV < 12.4%) and observer B (CoV < 18.8%). Regarding RV analyses, lowest interstudy variability was found for observer A (CoV < 10.7%) whilst fully automated analyses (CoV < 22.8) as well as observer B (CoV < 28.7%) demonstrated considerable inter-study variability.

**Table 2:** Interstudy reproducibility.

	Volumetric indices	Mean difference (SD of the Diff.)	ICC (95% CI)	CoV (%)
Experienced observer	LV mass (g/m^2^)	1.41 (5.35)	0.99 (0.98–1.00)	5.2
	LV EDV (ml/m^2^)	2.25 (6.51)	1.00 (0.99–1.00)	3.7
	LV ESV (ml/m^2^)	2.06 (8.01)	0.99 (0.98–1.00)	9.6
	LV SV (ml/m^2^)	0.33 (6.39)	0.98 (0.94–0.99)	7.0
	LV EF (%)	0.08 (2.98)	0.98 (0.94–0.99)	5.5
	RV EDV (ml/m^2^)	3.18 (6.72)	0.99 (0.96–1.00)	4.7
	RV ESV (ml/m^2^)	1.35 (5.97)	0.97 (0.92–0.99)	10.7
	RV SV (ml/m^2^)	4.02 (7.49)	0.93 (0.79–0.98)	8.7
	RV EF (%)	1.80 (3.51)	0.88 (0.67–0.96)	5.7
Inexperienced Observer	LV Mass (g/m^2^)	2.36 (15.78)	0.94 (0.83–0.98)	15.4
	LV EDV (ml/m^2^)	9.08 (23.76)	0.94 (0.83–0.98)	14.3
	LV ESV (ml/m^2^)	5.50 (14.69)	0.97 (0.91–0.99)	18.8
	LV SV (ml/m^2^)	3.58 (15.21)	0.86 (0.64–0.95)	17.2
	LV EF (%)	0.59 (5.40)	0.94 (0.84–0.98)	9.8
	RV EDV (ml/m^2^)	6.73 (17.70)	0.95 (0.86–0.98)	11.2
	RV ESV (ml/m^2^)	2.27 (16.29)	0.90 (0.73–0.96)	19.7
	RV SV (ml/m^2^)	4.45 (21.56)	0.73 (0.30–0.90)	28.7
	RV EF (%)	0.89 (10.50)	0.46 (0.00–0.80)	22.0
Automated	LV Mass (g/m^2^)	3.11 (7.55)	0.99 (0.97–1.00)	7.4
	LV EDV (ml/m^2^)	3.53 (14.09)	0.98 (0.95–0.99)	8.2
	LV ESV (ml/m^2^)	1.02 (9.94)	0.99 (0.97–1.00)	12.4
	LV SV (ml/m^2^)	2.50 (9.29)	0.93 (0.82–0.98)	10.2
	LV EF (%)	0.61 (3.01)	0.98 (0.94–0.99)	5.4
	RV EDV (ml/m^2^)	7.48 (22.12)	0.92 (0.78–0.97)	14.1
	RV ESV (ml/m^2^)	8.69 (13.07)	0.90 (0.65–0.97)	18.4
	RV SV (ml/m^2^)	1.26 (19.56)	0.77 (0.36–0.91)	22.8
	RV EF (%)	3.72 (7.12)	0.69 (0.20–0.88)	12.9

Interstudy variability for manual (experienced and inexperienced observer) as well as for fully automated assessments. LV mass is reported in gram, volumes in ml and EF in %.

*SD* standard deviation, *ICC* intraclass correlation coefficient, *CoV* coefficient of variation, *LV* left ventricular, *RV* right ventricular, *EDV* end-diastolic volume, *ESV* end-systolic volume, *SV*: stroke volume, *EF* ejection fraction.

For interobserver reproducibility, mean differences as well as corresponding SD, ICC and CoV of LV and RV volumes are reported in Table S1 (supplementary material) comparing automated with experienced and inexperienced manual analyses as well as comparing experienced and inexperienced manual analyses, showing overall excellent interobserver reproducibility of LV analyses (ICC 0.92–0.99) and fair to excellent reproducibility of RV metrics (ICC 0.43–0.97). Fully automated LV analyses shower better agreement with experienced than with inexperienced analyses. The automated algorithm overestimated RV EDV (mean difference 12.9 ± 13.8 ml/m^2^) and RV ESV (mean difference 10.5 ± 12.7 ml/m^2^) as compared to the experienced observer, while underestimated RV ESV (mean difference − 14.8 ± 9.0 ml/m^2^) as compared to the inexperienced observer.

### Sample size calculations

Sample sizes required for the detection of absolute changes in volumetric indices (10 g mass, 10 ml in volume or 5% EF) are reported in Table 3. Sample sizes were smallest for observer A, followed by fully automated analyses and largest for observer B. Whilst samples sizes of automated analyses for LV volumes were similar to those of observer A, sample sizes of automated analyses for RV volumes were similar to those of observer B. LV volume sample sizes ranged between n = 5 for LV mass and n = 11 for ESV for observer A, between n = 6 for EF and n = 32 for EDV for automated analyses and between n = 19 for EF to n = 89 for EDV for observer B. RV volume samples sizes ranged between n = 6 for ESV and n = 9 for SV for observer A, between n = 27 for ESV and n = 77 for EDV for automated analyses and between n = 42 for ESV and n = 73 for SV for observer B.

**Table 3:** Sample size calculation.

Detected differences in volumetric indices	Observer A		Observer B		Automated	
	SD	Sample size	SD	Sample Size	SD	Sample Size
**Left ventricle**						
10 g Mass	5.35	5	15.78	40	7.55	9
10 ml EDV	6.51	7	23.76	89	14.09	32
10 ml ESV	8.01	11	14.69	34	9.94	16
10 ml SV	6.39	7	15.21	37	9.29	14
5% EF	2.98	6	5.40	19	3.01	6
**Right ventricle**						
10 ml RV EDV	6.72	8	17.70	50	22.12	77
10 ml RV ESV	5.97	6	16.29	42	13.07	27
10 ml RV SV	7.49	9	21.56	73	19.56	61
5% RV EF	3.51	8	10.50	70	7.12	32

Sample size calculation for repeated measurements of left and right ventricular volumes for the detection of changes amounting to 10 g mass, 10 ml volumes and 5% EF respectively.

*EDV* end-diastolic volume, *ESV* end-systolic volume, *SV* stroke volume, *EF* ejection fraction, *SD* standard deviation.

## Discussion

The present study evaluates the interstudy variability of LV mass as well as LV and RV volumes quantified using a fully automated post-processing algorithm. Concerning LV analyses, the results demonstrate similarly high interstudy reproducibility of fully automated analyses as compared to an experienced CMR observer and show superior performance of fully automated analyses as compared to an inexperienced observer. In contrast, reliability of automated RV analyses is notably lower as compared to an experienced CMR observer.

CMR imaging represents the reference standard for the assessment of cardiac morphology and function due to a precise evaluation of bSSFP SA stacks covering the entire LV and RV^1^. However, in many departments CMR examinations are still not easily available since MR scanners are not always dedicated to CMR and consequently examinations and post-processing of the images are relatively time-consuming compared to other examinations. As a result cost-effectiveness is lower compared with competing methodology such as echocardiographic approaches even though CMR diagnostic information can often be considered of higher value^7,9^. Notwithstanding, mounting evidence emphasizes the need of CMR surveys in an increasing number of cardiac diseases^21^. To achieve high quality diagnostic examinations experience and training are important with a distinct effect on volumetric analyses and are particularly required in challenging anatomic conditions, e.g. patients with congenital heart disease^10,22^. User-independent fully automated assessments have been introduced for the evaluation of biventricular volumes showing promising results^11^. Machine learning and AI-based algorithms^23^ may indeed complement varying levels of user experience. Furthermore, process efficiency may be strengthened considering SA stacks volumetric analyses may be already performed parallel to scanning e.g. during LGE imaging, and thus might reduce analysis time and ultimately costs. Our results support a reliable use of fully automated LV analyses, showing objective and reproducible results.

Recently, automated analyses demonstrated feasibility and equally predictive prognostic value in 1017 patients following acute myocardial infarction compared to conventional analyses by trained and experienced medical personal^14^. Several previous studies applying the proposed automated algorithm showed consistently high interobserver reproducibility with experienced CMR observers^13,14^. The feasibility and reliability of automated LV analyses in clinical routine imaging is further underlined by the present data demonstrating high interstudy reproducibility. Future applications may expand to automated tissue characterisation e.g. scar quantification^14^ as well as deformation imaging^24^. Deformation imaging has gained recognition for enhanced risk prediction beyond conventional volumetric derived functional analyses, e.g. following acute myocardial infarction^25^ as well as ischemic and non-ischemic cardiomyopathy^26^. However, ongoing discussions about the reproducibility of deformation based approaches^27^ and limited data from large clinical train still hamper its unrestricted clinical use. At the current time, cardiac volumetric analyses still remain the gold-standard for quantitative functional assessments, despite its inability to assess regional function.

Guidelines for clinical decision making are inevitably based upon thresholds^28^. In certain clinical scenarios, decision making heavily relies on changes between serial examinations e.g. recovery of LVEF following acute myocardial infarction to evaluate implantable cardioverter defibrillator (ICD) therapy^3^. Serial examinations rely on the assumption that changes in cardiac mass and volumes are reliably detectable. However, most CMR imaging laboratories employ several CMR operators, often with different training experience, resulting in potential inter-observer variability if serial CMR examinations are analysed by different observers. This study confirms an overall excellent interstudy reproducibility for LV mass and volumes, best for manual assessments by an experienced observer and user-independent automated analyses and slightly lower for an inexperienced observer. Reproducibility of RV volumes was overall lower compared to LV metrics, which is in line with the available literature^6^. Whilst the experienced observer still achieved good to excellent reproducibility, variability between exams was high for the inexperienced observer. Automated assessments of RV volumes resulted in a slight improvement of reproducibility as compared to the inexperienced observer. We observed numerical differences for RV volumetry both for manual and automated analysis between the repeated exams. Even though they were statistically significant, their respective clinical relevance with a change of 2% in RV-EF should be interpreted with caution. On the other hand, defined cut-offs (e.g. for arrhythmogenic right ventricular cardiomyopathy (ARVC) end-diastolic volumes beyond 110 ml/m^2^ for male and 100 ml/m^2^ for female patients or an EF below 40%^29^) require precise volume assessments. Thus, inaccuracies in RV volume assessments bear potential clinical consequences. The present data support current evidence that precise and correct quantifications of RV metrics remain challenging and still require dedicated training which is probably due to the more complex anatomy of the RV as compared to the LV^10,30^. Because a strong link between RV functional but not structural changes with prognosis following acute myocardial infarction has been demonstrated^31^, the field of automated RV assessment and required analysis refinement and improvement warrants further investigation.

## Limitations

Sample size calculations and derived conclusions are based on n = 18 participants. Although reports indicate low sample sizes in CMR volume assessments^5^, statistical evaluations and generalisation may be limited. Detailed specifications of the automated algorithm that incorporates AI and deep learning models developed by the manufacturer are not disclosed; therefore, they cannot be described more precisely. The results of the study therefore apply to this specific cohort. Without knowing the exact types of scans used in the software’s training, it might be difficult to extrapolate the results to other cohorts, which should definitely be addressed in larger future studies. Furthermore, it will be interesting to address whether or not the results can be extrapolated to patients with a more demanding anatomy (e.g. patients with congenital heart disease).

## Conclusion

In this cohort, fully automated user-independent analyses allowed reliable serial investigations of LV volumes and function with comparably high interstudy reproducibility in relation to manual analyses performed by an experienced CMR observer. In contrast, fully automated RV assessments did not yet provide satisfying interstudy reproducibility and still require manual post-processing corrections by an experienced reader.

## Supplementary Information

Supplementary Information.

## Data Availability

Regarding data availability, we confirm that all relevant data are within the paper and all data underlying the findings are fully available without restriction from the corresponding author at the University Medical Centre Goettingen by researchers who meet the criteria for access to confidential data.

## References

[1] Pennell DJ (2010). Cardiovascular magnetic resonance. Circulation.

[2] Eitel I, de Waha S, Wöhrle J, Fuernau G, Lurz P, Pauschinger M (2014). Comprehensive prognosis assessment by CMR imaging after ST-segment elevation myocardial infarction. J. Am. Coll. Cardiol..

[3] Moss AJ, Zareba W, Hall WJ, Klein H, Wilber DJ, Cannom DS (2002). Prophylactic implantation of a defibrillator in patients with myocardial infarction and reduced ejection fraction. N. Engl. J. Med..

[4] White HD, Norris RM, Brown MA, Brandt PW, Whitlock RM, Wild CJ (1987). Left ventricular end-systolic volume as the major determinant of survival after recovery from myocardial infarction. Circulation.

[5] Grothues F, Smith GC, Moon JC, Bellenger NG, Collins P, Klein HU, Pennell DJ (2002). Comparison of interstudy reproducibility of cardiovascular magnetic resonance with two-dimensional echocardiography in normal subjects and in patients with heart failure or left ventricular hypertrophy. Am. J. Cardiol..

[6] Grothues F, Moon JC, Bellenger NG, Smith GS, Klein HU, Pennell DJ (2004). Interstudy reproducibility of right ventricular volumes, function, and mass with cardiovascular magnetic resonance. Am Heart J..

[7] Petitjean C, Dacher J-N (2011). A review of segmentation methods in short axis cardiac MR images. Med. Image Anal..

[8] Hautvast GLTF, Salton CJ, Chuang ML, Breeuwer M, O'Donnell CJ, Manning WJ (2012). Accurate computer-aided quantification of left ventricular parameters: experience in 1555 cardiac magnetic resonance studies from the Framingham Heart Study. Magn. Reson. Med..

[9] Axel L, Sodickson DK (2014). The need for speed: accelerating CMR imaging assessment of cardiac function. JACC Cardiovasc. Imaging..

[10] Beerbaum P, Barth P, Kropf S, Sarikouch S, Kelter-Kloepping A, Franke D (2009). Cardiac function by MRI in congenital heart disease: impact of consensus training on interinstitutional variance. J. Magn. Reson. Imaging..

[11] van Geuns RJM, Baks T, Gronenschild EHBM, Aben J-PMM, Wielopolski PA, Cademartiri F, de Feyter PJ (2006). Automatic quantitative left ventricular analysis of cine MR images by using three-dimensional information for contour detection. Radiology.

[12] Queirós S, Barbosa D, Engvall J, Ebbers T, Nagel E, Sarvari SI (2016). Multi-centre validation of an automatic algorithm for fast 4D myocardial segmentation in cine CMR datasets. Eur. Heart J. Cardiovasc. Imaging..

[13] Backhaus SJ, Staab W, Steinmetz M, Ritter CO, Lotz J, Hasenfuß G (2019). Fully automated quantification of biventricular volumes and function in cardiovascular magnetic resonance: applicability to clinical routine settings. J. Cardiovasc. Magn. Reson..

[14] Schuster A, Lange T, Backhaus SJ, Strohmeyer C, Boom PC, Matz J (2020). Fully automated cardiac assessment for diagnostic and prognostic stratification following myocardial infarction. J. Am. Heart Assoc..

[15] Kramer CM, Barkhausen J, Flamm SD, Kim RJ, Nagel E (2013). Standardized cardiovascular magnetic resonance (CMR) protocols 2013 update. J. Cardiovasc. Magn. Reson..

[16] Schulz-Menger J, Bluemke DA, Bremerich J, Flamm SD, Fogel MA, Friedrich MG (2013). Standardized image interpretation and post processing in cardiovascular magnetic resonance: Society for Cardiovascular Magnetic Resonance (SCMR) board of trustees task force on standardized post processing. J. Cardiovasc. Magn. Reson..

[17] Schulz-Menger J, Bluemke DA, Bremerich J, Flamm SD, Fogel MA, Friedrich MG (2020). Standardized image interpretation and post-processing in cardiovascular magnetic resonance—2020 update: Society for Cardiovascular Magnetic Resonance (SCMR): Board of Trustees Task Force on Standardized Post-Processing. J. Cardiovasc. Magn. Reson..

[18] Suinesiaputra A, Bluemke DA, Cowan BR, Friedrich MG, Kramer CM, Kwong R (2015). Quantification of LV function and mass by cardiovascular magnetic resonance: multi-center variability and consensus contours. J. Cardiovasc. Magn. Reson..

[19] Morton G, Schuster A, Jogiya R, Kutty S, Beerbaum P, Nagel E (2012). Inter-study reproducibility of cardiovascular magnetic resonance myocardial feature tracking. J. Cardiovasc. Magn. Reson..

[20] Bland M, Altman D (1986). Statistical methods for assessing agreement between two methods of clinical measurement. Lancet..

[21] von Knobelsdorff-Brenkenhoff F, Schulz-Menger J (2016). Role of cardiovascular magnetic resonance in the guidelines of the European Society of Cardiology. J. Cardiovasc. Magn. Reson..

[22] Karamitsos TD, Hudsmith LE, Selvanayagam JB, Neubauer S, Francis JM (2007). Operator induced variability in left ventricular measurements with cardiovascular magnetic resonance is improved after training. J. Cardiovasc. Magn. Reson..

[23] Leiner T, Rueckert D, Suinesiaputra A, Baeßler B, Nezafat R, Išgum I, Young AA (2019). Machine learning in cardiovascular magnetic resonance: basic concepts and applications. J. Cardiovasc. Magn. Reson..

[24] Backhaus SJ, Metschies G, Zieschang V, Erley J, Mahsa Zamani S, Kowallick JT (2021). Head-to-head comparison of cardiovascular MR feature tracking cine versus acquisition-based deformation strain imaging using myocardial tagging and strain encoding. Magn. Reson. Med..

[25] Eitel I, Stiermaier T, Lange T, Rommel K-P, Koschalka A, Kowallick JT (2018). Cardiac magnetic resonance myocardial feature tracking for optimized prediction of cardiovascular events following myocardial infarction. JACC Cardiovasc. Imaging..

[26] Romano S, Judd RM, Kim RJ, Kim HW, Klem I, Heitner JF (2018). Feature-tracking global longitudinal strain predicts death in a multicenter population of patients with ischemic and nonischemic dilated cardiomyopathy incremental to ejection fraction and late gadolinium enhancement. JACC Cardiovasc. Imaging.

[27] Schuster A, Hor KN, Kowallick JT, Beerbaum P, Kutty S (2016). Cardiovascular magnetic resonance myocardial feature tracking: concepts and clinical applications. Circ. Cardiovasc. Imaging..

[28] Ponikowski P, Voors AA, Anker SD, Bueno H, Cleland JGF, Coats AJS (2016). 2016 ESC Guidelines for the diagnosis and treatment of acute and chronic heart failure: The Task Force for the diagnosis and treatment of acute and chronic heart failure of the European Society of Cardiology (ESC) developed with the special contribution of the Heart Failure Association (HFA) of the ESC. Eur. Heart J..

[29] Marcus FI, McKenna WJ, Sherrill D, Basso C, Bauce B, Bluemke DA (2010). Diagnosis of arrhythmogenic right ventricular cardiomyopathy/dysplasia: proposed modification of the Task Force Criteria. Eur Heart J..

[30] Hudsmith L, Petersen S, Francis J, Robson M, Neubauer S (2005). Normal human left and right ventricular and left atrial dimensions using steady state free precession magnetic resonance imaging. J. Cardiovasc. Magn. Reson..

[31] Stiermaier T, Backhaus SJ, Matz J, Koschalka A, Kowallick J, de Waha-Thiele S (2020). Frequency and prognostic impact of right ventricular involvement in acute myocardial infarction. Heart.

