# Supplementary material for: Impact of fully automated assessment on interstudy reproducibility of biventricular volumes and function in cardiac magnetic resonance imaging
Source: Sci Rep. 2021 Jun 2;11:11648. doi: 10.1038/s41598-021-90702-9 (PMC8172876; doi:10.1038/s41598-021-90702-9)
Supplement: Supplementary file 1 — Supplementary Information. [file 41598_2021_90702_MOESM1_ESM.docx]

**Supplementary material**

|  | Volumetric indices | Mean Difference  (SD of the Diff.) | ICC (95% CI) | CoV (%) |
| --- | --- | --- | --- | --- |
| Automated vs. Experienced Observer | LV Mass (g/m²) | -0.59 (9.03) | 0.99 (0.96-1.00) | 8.7 |
|  | LV EDV (ml/m²) | -5.98 (8.23) | 0.99 (0.96-1.00) | 4.8 |
|  | LV ESV (ml/m²) | -4.49 (7.22) | 0.99 (0.97-1.00) | 8.8 |
|  | LV SV (ml/m²) | -1.55 (7.78) | 0.96 (0.90-0.99) | 8.6 |
|  | LV EF (%) | 0.89 (3.53) | 0.97 (0.91-0.99) | 6.5 |
|  | RV EDV (ml/m²) | 12.93 (13.82) | 0.92 (0.52-0.98) | 9.4 |
|  | RV ESV (ml/m²) | 10.53 (12.67) | 0.84 (0.33-0.95) | 20.6 |
|  | RV SV (ml/m²) | 1.87 (10.66) | 0.90 (0.73-0.96) | 12.5 |
|  | RV EF (%) | -3.12 (7.32) | 0.61 (0.02-0.85) | 12.5 |
| Automated vs. Inexperienced Observer | LV Mass (g/m²) | 2.09 (19.13) | 0.92 (0.78-0.97) | 18.7 |
|  | LV EDV (ml/m²) | 7.69 (13.95) | 0.97 (0.91-0.99) | 8.4 |
|  | LV ESV (ml/m²) | 4.44 (9.10) | 0.98 (0.95-0.99) | 11.7 |
|  | LV SV (ml/m²) | 3.18 (8.87) | 0.95 (0.87-0.98) | 10.0 |
|  | LV EF (%) | 0.15 (3.67) | 0.97 (0.92-0.99) | 6.7 |
|  | RV EDV (ml/m²) | -1.44 (13.74) | 0.97 (0.91-0.99) | 8.9 |
|  | RV ESV (ml/m²) | -14.78 (8.99) | 0.88 (0.00-0.97) | 12.1 |
|  | RV SV (ml/m²) | 13.31 (16.72) | 0.74 (0.15-0.91) | 21.0 |
|  | RV EF (%) | 9.90 (7.90) | 0.50 (0.00-0.82) | 15.1 |
| Inexperienced vs. Experienced observer | LV Mass (g/m²) | -2.68 (13.10) | 0.96 (0.88-0.98) | 12.9 |
|  | LV EDV (ml/m²) | -13.67 (16.17) | 0.96 (0.75-0.99) | 18.7 |
|  | LV ESV (ml/m²) | -8.94 (12.61) | 0.97 (0.86-0.99) | 16.7 |
|  | LV SV (ml/m²) | -4.73 (10.83) | 0.93 (0.81-0.98) | 12.5 |
|  | LV EF (%) | 0.74 (5.22) | 0.94 (0.85-0.99) | 9.5 |
|  | RV EDV (ml/m²) | 14.36 (17.06) | 0.90 (0.50-0.97) | 11.0 |
|  | RV ESV (ml/m²) | 25.31 (12.49) | 0.67 (0.00-0.91) | 15.3 |
|  | RV SV (ml/m²) | -11.45 (14.90) | 0.76 (0.21-0.92) | 20.4 |
|  | RV EF (%) | -13.02 (6.98) | 0.43 (0.00-0.80) | 14.8 |
| **Table S1 Interobserver reproducibility** Interobserver variability based on segmentation results of Exam 1. LV mass is reported in gram, volumes in ml and EF in %. SD: standard deviation, ICC: intraclass correlation coefficient, CoV: coefficient of variation, LV: left ventricular, RV: right ventricular, EDV: end-diastolic volume, ESV: end-systolic volume, SV: stroke volume, EF: ejection fraction | | | | |
